# Emergency care of traumatic brain injuries in Pakistan: a multicenter study

**DOI:** 10.1186/1471-227X-15-S2-S12

**Published:** 2015-12-11

**Authors:** Junaid Ahmad Bhatti, Kent Stevens, Muhammad Umer Mir, Adnan A Hyder, Junaid Abdul Razzak

**Affiliations:** 1 Department of Emergency Medicine, Aga Khan University, Karachi, Pakistan; 2 Sunnybrook Health Sciences Centre Research Institute, Toronto, Canada; 3 Institute for Clinical Evaluative Sciences, Toronto, Canada; 4 University of Toronto, Department of Surgery, Toronto, Canada; 5 Department of Surgery, John Hopkins University School of Medicine, Baltimore, Maryland, United States; 6 Johns Hopkins International Injury Research Unit, Department of International Health, Johns Hopkins Bloomberg School of Public Health, Baltimore, Maryland, USA; 7 Global Health Systems & Development, Tulane University School of Public Health, New Orleans, Louisiana, USA; 8 Department of Emergency Medicine, John Hopkins School of Medicine, Baltimore, Maryland, United States; 9 The author was affiliated with the Department of Emergency Medicine, Aga Khan University, Karachi, Pakistan at the time when study was conducted

**Keywords:** Access to care, economic costs, traumatic brain injury, Pakistan

## Abstract

**Background:**

This study assessed factors associated with emergency care outcomes and out-of-pocket treatment costs in traumatic brain injury (TBI) patients in Pakistan.

**Methods:**

Data on TBI patients were extracted from a four-month surveillance study conducted in the emergency departments (ED) of seven large teaching hospitals. Emergency care access to physicians and imaging facilities were compared with respect to ED outcomes (discharged, admitted or dead). Out-of-pocket treatment costs (in United States dollars [USD]) were compared among different patient strata.

**Results:**

ED outcomes were available for 1,787 TBI patients. Of them, most were males (79%), aged <25 years (46%) and arrived by ambulances (32%). Nurses or paramedical staff saw almost all patients (95%). Physicians with practice privileges (medical officers, residents or consultants) saw about half (55%) of them. Computerized tomography (CT) scans were performed in two of five patients (40%). Of all, 26% (n = 460) were admitted and 3% died (n = 52). Emergency care factors significantly associated with being admitted or died were arriving by ambulance (adjusted odds ratio [aOR] = 2.37, 95% confidence interval (CI) [95%CI] = 1.78-3.16); seen by medical officer/residents (aOR = 2.11; 95%CI = 1.49-2.99); and had CT scan (aOR = 2.93; 95%CI = 2.25-3.83). Out-of-pocket treatment costs at the ED were reported in 803 patients. Average costs were USD 8, (standard deviation [SD] = 23). Costs were twice as high in those arriving in ambulances (USD 20, SD = 49) or who underwent CT scans (USD 16, SD = 37).

**Conclusion:**

TBI patients' access to ambulance transport, experienced physicians, and imaging facilities during emergency care needs to be improved in Pakistan.

## Background

Emergency care plays a pivotal role in any healthcare system [1]. Effective emergency care systems can significantly reduce the risks of mortality and morbidity [2]. For example, emergency care outcomes for similar severe injuries can be significantly worse in low- and middle-income countries (LMICs) than in high-income countries (HICs) [2,3]. Inadequate human and material resources in LMICs are considered as reasons for such differences [2]. Presently, there are few studies on the emergency care quality in treating severe injuries [4]. The World Health Organization (WHO) resolution 60.22 calls all member nations to conduct evaluations in order to inform priorities in emergency care [5].

Traumatic brain injuries (TBIs) - defined as a disruption in brain functioning resulting from sudden, unexpected, intolerable application of mechanical force - are relatively common and often require specialized emergency care [6,7]. The nature of TBIs are usually described as either "head injury/contusion," "intracranial bleed" or "concussion" [8]. During 2002-2006, TBIs were the leading cause of death and disabilities in the United States (US) [9]. It was estimated that TBIs account for over 1.7 million emergency department (ED) visits and over fifty thousand deaths annually in the US. The situation in LMICs is similar, as TBIs were observed in more than 75% of fall-related injuries and 50% of road traffic injuries [10]

An early identification of TBIs and appropriate emergency care can prevent long-term disabilities [11]. While emergency care of TBIs in HICs, including the US, has seen significant improvements over the last four decades [6,12], limited information is available about emergency care of TBIs in LMICs [10,13]. Several pieces of information are necessary for determining whether TBI care access is appropriate [11]. Primarily, these include availability of specialized human (e.g., experienced physicians) and material resources (e.g., ambulance and computerized tomography [CT] scan) for patients with moderate and severe TBIs [14]. The cost of TBIs is another consideration in these evaluations [15]. An Australian study found that the ED care costs for TBI patients were significantly higher than the average ED care costs [16]. A Vietnamese study showed that low-income households faced difficulties in affording TBI care [17].

Pakistan is low-income country with over 180 million population, with a high rate of TBIs [18,19]. A large road traffic injury surveillance study (n>100,000) in Pakistan showed that nearly a third of patients had a TBI, and of them about 10% percent had moderate to severe TBI [18]. Despite the noted burden, no information is available about the emergency care of TBI patients in Pakistan [19-21]. This study evaluated factors associated with emergency care outcomes and out-of-pocket treatment costs in TBI patients in Pakistan [22-24].

## Methods

### Study Design

We studied TBI patients as part of a multicenter pilot surveillance study - the Pakistan National Emergency Departments Surveillance Study (hereafter referred as Pak-NEDS). More details on the aims and design of Pak-NEDS are available elsewhere [22]. In brief, this study assessed both the feasibility of establishing an ED surveillance system and the level of emergency care at teaching hospitals in Pakistan. EDs of seven teaching hospitals in four different provinces of Pakistan participated in this study, including the Aga Khan University (AKU) and Jinnah Post-graduate Medical Center in Karachi, Benazir Bhutto Hospital in Rawalpindi, Lady Reading Hospital in Peshawar, Mayo Hospital in Lahore, Sandeman Provincial Hospital in Quetta, and Shifa International Hospital in Islamabad.

All selected hospitals had 24-hour EDs with nurses, physicians and support staff. As all were teaching hospitals, there were three levels of physicians, junior in-training doctors (or house physicians), medical officer or specialty residents (experienced), and consultants. Junior physicians had limited privileges for practice, whereas medical officers, residents and consultants had complete privileges. All patients were usually required to be seen by a physician with full privileges, which was not always possible when there was a high patient volume. Except for Benazir Bhutto Hospital in Rawalpindi, all hospitals had an in-house neurosurgery department with specialized in-patient service. AKU and Shifa International Hospital were private sector hospitals requiring all expenses to be paid by patients or their insurance agencies. The remaining hospitals had no physician charges, but patients could be required to pay up-front and out-of-pocket for medications or examinations, including imaging that was not covered by the government.

AKU was the main coordinating center for the study. Ethical approval was obtained from all participating sites. Pak-NEDS was conducted for a four month period per selected site from Nov 2010 to Mar 2011. All patients (all ages) presenting to the ED during the above period were eligible for recruitment in Pak-NEDS.

### Measures

A one-page standardized tool was developed based on the National Hospital Ambulatory Medical Care Survey (NHAMCS) tool of the Centers for Disease Control and Prevention, USA [25], which was modified in consultation with emergency medicine experts from the participating institutes to ensure compatibility with the local ED settings [23,26,27]. The tool included questions related to patient characteristics such as age and gender, as well as hospital, presenting complaints, health professional evaluating patients, and treatment provided, including imaging (e.g., X-ray, CT scan) in the ED. The tool included information about outcomes at the emergency department such as whether a patient was discharged from the ED, admitted to hospital or died. At the end of the questionnaire, the respondents were also asked whether they had any out-of-pocket expenses during the course of their ED treatment, including transport charges. All of these expenses were recorded in Pakistani rupee (PKR).

### Procedures

Study-specific staff with previous medical care experience worked in three shifts to collect data on the designated tool. Twenty-four hour data collection was conducted on all sites except one; there the staff worked in two eight-hour shifts every day. At this site, the shifts were rotated every week to have a sample of patients presenting during the 24 hours. Either the patient or their next of kin or guardian was interviewed in the ED, along with a review of the ED records in order to collect the required information. Hard copies of the data collection tool were sent to the coordinating center at AKU on a weekly basis. Data were entered at AKU using EpiInfo version 3.3.2. A research coordinator supervised the data entry procedures.

### Patient selection

For this study, patients were selected when presenting complaints that were described as "head injury" and the nature of injury was one of the TBI-specified above (see introduction). Only new patients were included [28].

### Analyses

Two analyses were conducted. In the first one, patients with complete data on their ED outcomes were included (n = 1,787). Emergency care factors associated with worse ED outcomes, i.e., either admission or death, were assessed using the chi-square test. We also constructed a multivariate logistic regression model in which only variables which had a strong association with some ED outcomes (*P*≤0.10) in the univariate analysis were kept in the final model. In the second analysis, we included only those patients where out-of-pocket treatment costs were available (n = 803). The association of patient factors with out-of-pocket costs was assessed using one-way ANOVA test. The *P*<0.05 indicated that at least one patient strata had significantly higher or lower costs than others. All costs were in United States dollars (USD) with the conversion rate of 100 Pakistani rupees ~ 1 USD. All analyses were performed on SPSS, version 19.

## Results

**Figure 1:**
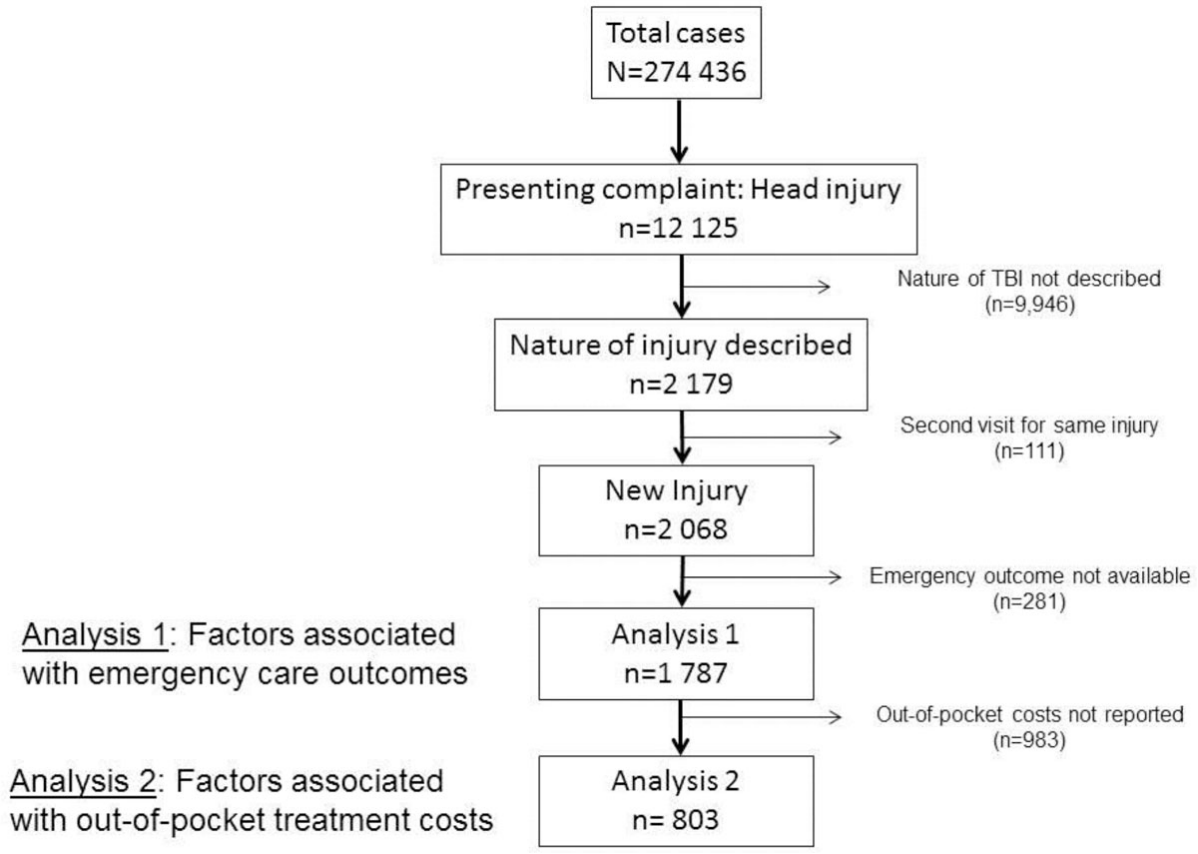
**Selection of traumatic brain injury patients for analyses**.

Figure 1 describes the selection of patients for proposed analysis. A total of 274,436 patients were included in Pak-NEDS. Of these, nearly 12,125 patients had presenting complaints as TBI, but the nature of the TBI was documented in only 2,179 patients. After excluding patients with missing ED outcomes, 1,787 patients were considered in the ED outcome analysis. Over three-quarters (79.3%) of patients were males and almost half (46.4%) were aged < 25 years (Table 1). Most TBIs (63.5%) were unintentional injuries, and the remaining one-third (36.4%) were intentional. Most intentional TBIs were assaults (88.1%, n = 553), and the rest were self-harm injuries (12.9%, n = 82). Mechanisms of TBI were reported in 370 patients (20.7%), in whom the most common were road traffic crashes (48.6%) followed by falls (22.4%). Only a third (31.6%) of TBI patients arrived by ambulances, whereas over half of them (53.1%) arrived by other transport. Nearly all TBI patients (98.5%) came to public hospitals. Nurses or paramedics saw almost all TBI patients (95.2%); junior doctors (house physicians) saw more than half (66.3%), and medical officers and residents saw about half of them (54.7%). Consultant physicians saw only one percent of the TBI patients in the ED. The Glasgow Coma Scale (GCS) (for TBI severity) was reported in only 9% of patients (n = 162). Two in five patients (39.5%) underwent a CT scan. Similarly, x-rays were performed in about half of the patients (45.2%).

**Table 1:** Characteristics and outcomes of Traumatic Brain Injury Patients in Pakistan

	Total	Discharged from ED	Admitted	Died	P*	Odds ratio for admission or death	95% Confidence Interval
	(n)	(n)	(n)	(n)			
	%	%	%	%			
**Total n**	(n = 1787)	(n = 1275)	(n = 460)	(n = 52)			
							
**Age (in years)**	(n = 1650)	(n = 1170)	(n = 432)	(n = 48)	0.10		
- 0 - 14	15.8	21.7	16.9	27.1		0.67	0.43-1.05
- 15 - 24	25.8	26.7	24.3	16.7		0.81	0.53-1.23
- 25 - 44	39.6	38.5	42.4	39.6		0.94	0.64-1.39
- 45+	14.1	13.1	16.4	16.7		1	
**Gender**	(n = 1771)	(n = 1262)	(n = 458)	(n = 51)	0.09		
- Female	20.7	21.9	17.9	13.7		1	
- Male	79.3	78.1	82.1	86.3		1.20	0.86-1.68
**Intent of injury**	(n = 1746)	(n = 1260)	(n = 436)	(n = 51)	0.01		
- Unintentional	63.6	65.3	58.0	68.6		1	
- Intentional	36.4	34.7	42.1	31.4		1.89	1.41-2.53
**Injury mechanism**	(n = 370)	(n = 267)	(n = 95)	(n = 8)	0.68		
- Road traffic crash	48.6	49.1	47.4	50.0			
- Falls	22.4	24.0	18.9	12.5			
- Animal bite or attack	6.2	6.7	4.2	12.5			
- Struck by an object	4.3	3.7	6.3	0			
- Others	18.4	16.5	23.2	25.0			
**Arriving by ambulance**	(n = 1738)	(n = 1248)	(n = 438)	(n = 52)	<0.001		
- No	68.4	74.7	52.3	53.8		1	
- Yes	31.6	25.3	47.7	46.2		2.37	1.78-3.16
**Hospital**	(n = 1787)	(n = 1275)	(n = 460)	(n = 52)	0.006		
- Public	98.5	99.1	97.0	100.0		1	
- Private	1.5	0.9	3.0	0		0.46	0.10-2.10
**Care providers**							
Nurse or paramedic	95.2	95.1	95.2	96.2	0.95		
- Interns	66.3	77.5	38.9	32.7	<0.001	0.17	0.12-0.23
- Medical officer or residents	54.7	49.4	82.6	92.3	<0.001	2.11	1.49-2.99
- Attending physician	1.0	1.0	0.9	0	0.74		
**Radiography**							
- Computerized tomography	39.5	31.0	63.0	34.6	<0.001	2.93	2.25-3.83
- X-rays	45.2	41.2	58.9	21.2	<0.001	1.13	0.87-1.48

A <0.05 indicated that at least one patient strata was different from others in terms of proportion of admissions and deaths.

ED - Emergency Department

Table 1 also shows the distribution of patients with respect to ED outcomes. Of the total number of patients, 1,275 (71.3%) were discharged from the ED, 460 (25.8%) were admitted and 52 died (2.9%). Ambulance use was almost twice as high (P < 0.001) in patients who were admitted (47.7%) or who died (46.2%) as compared to those who were discharged (25.3%). The likelihood of being seen by medical officers and residents was twice (*P *< 0.001) as high in those who were admitted (82.6%) than those who were discharged from ED (49.4%). The proportion of those who had a CT scan was significantly higher (P < 0.001) in those who were admitted (63.0%) than those who were discharged from the ED (31.0%). Similarly, the proportion of x-rays performed was significantly higher (P < 0.001) in those who were admitted (58.9%) than those who were discharged from the ED (41.2%). Logistic regression analysis showed that TBI-related hospital admissions or deaths were associated with intentional TBIs (adjusted odds ratio [aOR] = 1.89), ambulance use (aOR = 2.37), examination by medical officers/residents (aOR = 2.11), and CT scan (aOR = 2.93)

Out-of-pocket-treatment costs were reported for 803 patients (44.9%) (Table 2). On average, a TBI patient spent about USD 8.4 (SD = 23.8) during the emergency care. Significantly higher costs (*P*<0.001) were noted for those who were later admitted (USD 18.2, SD = 40.3) or who died (USD 21.6, SD = 25.2) in the ED than those who were discharged (USD 3.3, SD = 5.4). Similarly, those arriving in ambulances paid a significantly higher cost (USD 20.4, SD = 49.0) than those who used other means (USD 2.6, SD = 4.2). Those who had a CT scan spent significantly more (USD 16.1, SD = 36.7, *P*<0.001) than those who did not have a CT scan.

**Table 2:** Out-of-pocket treatment costs (US$) for traumatic brain injury patients

	Patient (n)	Average expenses (in US$)	Standard deviation	P*
**Total**	803	8.39	23.75	
				
**Outcome**				<0.001
- Discharged	536	3.27	5.35	
- Admitted	229	18.17	40.33	
- Died	38	21.64	25.20	
**Age**				0.05
0-14 years	202	8.94	16.57	
15-24 years	206	5.93	11.53	
25 - 44 years	260	8.20	19.79	
45 +years	105	14.00	50.19	
**Gender**				0.06
Male	608	9.20	26.33	
Female	186	5.94	12.87	
**Intent of injury**				0.25
- Unintentional	637	8.71	24.91	
- Intentional	102	6.15	24.93	
**Arrived by ambulance**				<0.001
- Yes	135	20.37	48.99	
- No	633	2.62	4.20	
**Care providers**				
- Nurse or paramedic	755	8.48	24.20	0.65
- Junior in training doctor	354	2.82	11.12	<0.001
- Medical officer or residents	713	8.93	25.06	0.07
**Radiology**				
- Computerized tomography	267	16.11	36.70	<0.001
- X-rays	479	6.81	14.61	0.02

* P < 0.05 signifies that average costs were significantly different from others in at least one patient strata

## Discussion

To the best of our knowledge, Pak-NEDS is the largest multicenter study of emergency patients in Pakistan. Some findings of this study were consistent with previous ones from neurosurgery centers showing that young men, traffic crashes, and falls were associated with TBIs in Pakistan [19,21]. This study showed that access to attending physicians at the level of ED is only one percent. Similarly, a significant proportion of those who were admitted did not have CT scans performed at the ED. Out-of-pocket treatment costs were significantly higher than average for those undergoing CT scans or those using ambulances.

This study observed a higher intensity of ambulance use with more severe TBIs. For example, TBI injury severity increased ambulance use: 66% of severe TBI and 56% of moderate TBI (as determined by the GCS) arrived by ambulances (results not shown because of <10% documentation) [29]. Compared to the statistics from settings with resources, ambulance use among TBI patients was still low in Pakistan [30,31]. Of note, ambulance services in Pakistan suffer from material and human resource constraints [29,32]. Further, the paramedics may not be fully trained to provide adequate care to TBI patients during transport [23,26]. Taken together, this study suggested improving access to quality prehospital care of TBI patients in Pakistan.

The findings pointed out that the availability and costs of imaging might also be important constraints for medical care of TBIs in Pakistan. CT scan machines have considerable maintenance costs, and these are often transferred to patients as fees-for-use. As about two-thirds of the Pakistani population earn less than US$2 a day per head, most TBI patients might not be able to afford imaging unless they use their savings or borrow funds [24]. These circumstances might explain why a third of admitted patients did not have CT scans in the ED [10,33]. The high costs for CT imaging might have clinical implications, as it would be difficult for physicians to follow international guidelines for TBI management in Pakistan. The feasibility of the latter is supported by implementation research from the Brain Trauma Foundation guidelines (including CT availability) in a private hospital: an experience that had a positive impact on reducing inpatient mortality and length of stay [34]. Clearly, there is a need to establish and monitor practice benchmarks of emergency care of TBIs in Pakistan as well as ensuring that low-income strata have access to imaging facilities when needed.

This study may have several limitations. A large number of patients had to be excluded from analysis because information about the nature of their TBI was not available. The data collectors were dependent on physicians to document this information. Still, this study had a considerable sample of TBI patients to assess factors associated with TBI care. Similarly, information about several factors, including the TBI mechanism and severity was not documented. For example, the Glasgow Coma Scale was reported in only one in ten patients. Relatedly, this study might also underestimate minor TBIs or concussions, most of which usually go unrecognized [35]. Further, the results of the ED outcomes might be biased towards admitted patients as there were fewer deaths. Lastly, it is likely that out-of-pocket treatment costs might have been underestimated in some circumstances. For example, this analysis might not have accounted for expenses billed at discharge or those incurred during transport to home.

## Conclusion

These findings suggest several areas of improving TBI care in Pakistan. The potential interventions might include increased access to ambulance services, better reporting of TBI details, increased examination by experienced physicians in the ED, and the availability of imaging in the ED of tertiary care centers. These steps need to be taken in conjunction with other general measures for emergency TBI care, such as improving public awareness about TBI care and capacity building within the healthcare system.

## Competing interests

The authors declare that they have no competing interests.

## Authors' contributions

JAB the data analysis and the initial draft. AAH and JAR conceptualized Pak-NEDS, supervised data collection, contributed in the analyses and manuscript writing. NS, KS and MUM contributed in the data cleaning, analysis, and interpretation. All authors critically read and approved the final draft before submission.
